# Clinical prediction model and 2-year mortality for multiple organ dysfunction in patients aged 80 years or older following hip fracture surgery: a prospective cohort study

**DOI:** 10.3389/fmed.2025.1515557

**Published:** 2025-07-25

**Authors:** Lei Liu, Yang Deng, Zhijun Qin, Ying Zhang, Xi Yang, Ji Feng, Chenzhu Yin

**Affiliations:** ^1^Hospital Infection Management Department, Sichuan Province Orthopedic Hospital, Chengdu, China; ^2^Intensive Care Unit, Sichuan Province Orthopedic Hospital, Chengdu, China; ^3^Anesthesiology Department, Sichuan Province Orthopedic Hospital, Chengdu, China

**Keywords:** elderly, hip fracture surgery, multiple organ dysfunction syndrome, mortality, prediction model

## Abstract

**Background:**

The prediction of postoperative complications is vital in the management of hip fracture. This study specifically examined the occurrence of multiple organ dysfunction syndrome in the elderly (MODSE) following hip fracture surgery and explored its predictive model and correlation with long-term mortality.

**Methods:**

This prospective cohort study included all patients aged 80 years and older who underwent hip fracture surgery at a tertiary orthopedic hospital between January 2020 and March 2021. The patients were categorized into the MODSE and non-MODSE groups. The pre, peri, and postoperative variables were retrospectively screened to establish and validate prediction model for MODSE. The patients were subsequently followed up prospectively until 2 years after discharge in order to explore the association between MODSE and long-term mortality.

**Results:**

Significant discrepancies in clinical characteristics were observed between MODSE and non-MODSE patients. Those with a preoperative age-adjusted Charlson Comorbidity Index > 5, Impaired swallowing, an Acute Physiology and Chronic Health Evaluation II score ≥ 12 within the initial 24 h post-surgery, prothrombin time > 14 s, along with high-sensitivity cardiac troponin T > 14 pg./mL and procalcitonin levels > 0.25 ng/mL on the first day after surgery were found to have a higher likelihood of developing MODSE. Moreover, the presence of MODSE correlated with a 3.13-fold and 2.88-fold increased risk of mortality at 1 and 2 years post-discharge, respectively.

**Conclusion:**

Predicting postoperative MODSE in elderly hip fracture patients is essential and feasible, as its occurrence represents poor outcome during hospitalization and predicts heightened long-term mortality rates.

## 1 Introduction

Hip fracture stands out as a predominant traumatic disease among the elderly, attributable to aging, bone degeneration, and accidental fall. With the accelerated pace of global aging, the incidence of hip fracture has surged, emerging as a global health concern (1, 2). China faces an impending “aging tsunami” mirroring global trends (3). Notably, the majority of hip fracture cases in urban China were found to affect individuals aged 75 years and older, with those aged 85 years and older representing 11.5% of the total cases (4). The aftermath of hip fracture is profound, often leading to post-fracture disabilities and alarmingly high 1-year mortality rates ranging from 20 to 30%, rendering it a devastating event for the elderly population (5, 6).

Elderly hip fracture patients frequently encounter a variety of postoperative complications. These may include pneumonia, hypotension, gastrointestinal hemorrhage, stroke, acute renal failure (7, 8), and, in severe cases, potentially life-threatening multiple organ dysfunction syndrome (MODS). There is no doubt that the vulnerability of elderly patients with hip fracture has significantly lowered the threshold for developing MODS. Chinese scholars have formulated diagnostic criteria for multiple organ dysfunction in the elderly (MODSE) based on the characteristics of elderly patients (Supplementary Table S1) (9, 10). Unlike the organ dysfunction scoring systems typically employed in intensive care unit (such as Sequential Organ Failure Assessment, Logistic Organ Dysfunction System, and Multiple Organ Dysfunction Score) (11), the MODSE diagnostic criteria include a broader range of organs or systems and their corresponding indicators. Furthermore, within these diagnostic criteria, organ dysfunction is classified into stages of pre-failure and failure. This could facilitate early detection of organ dysfunction in vulnerable patients after experiencing a hip fracture. Nonetheless, there has been no systematic research specifically investigating MODSE following hip fracture.

Our hypothesis is that elderly patients with hip fracture are at high risk of developing MODSE, and that the presence of MODSE during hospitalization may be associated with long-term prognosis. The aim of this study was to investigate the risk factors and outcomes of MODSE following hip fracture surgery in patients aged 80 years and older.

## 2 Methods

### 2.1 Study design and ethical approval

This prospective cohort study was conducted at a tertiary orthopedic hospital. Ethical approval (KY2020-032-01) was obtained from the Ethical Committee of the hospital on 3 November 2020, in accordance with the Declaration of Helsinki. Written informed consent was secured from all participants or their legally authorized representatives. The study was prospectively registered with the Chinese Clinical Trial Registry (ChiCTR2000038747, 30 September 2020).

### 2.2 Participant selection

#### 2.2.1 Inclusion criteria

Age ≥80 years, femoral neck or intertrochanteric fracture, surgical treatment between 1 January 2020 and 31 March 2021.

#### 2.2.2 Exclusion criteria

Open/pathologic fractures, multisite fractures or concurrent organ trauma, periprosthetic fractures or fractures >14 days.

**Figure 1:**
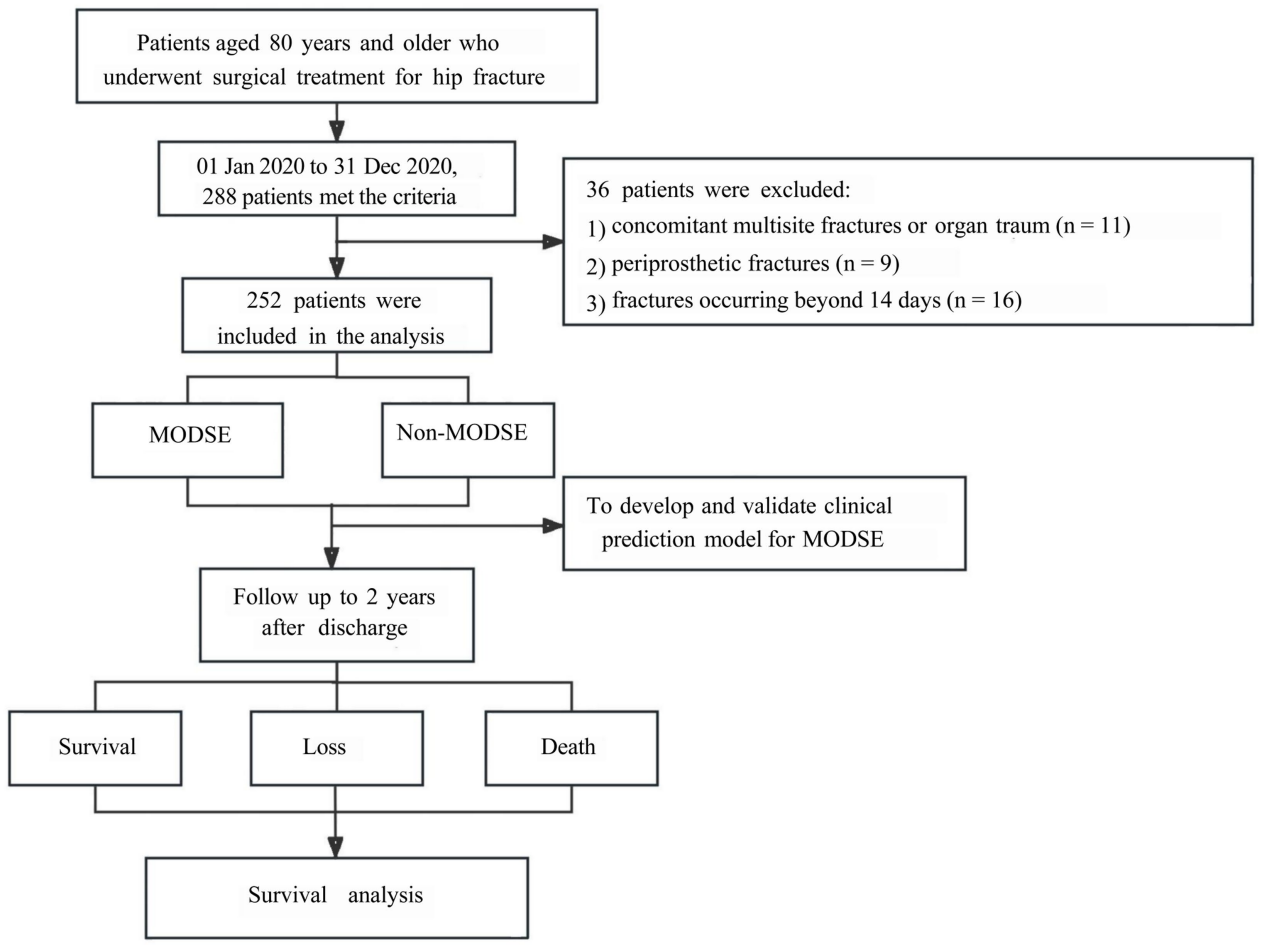
Flow chart of study participants. MODSE, multiple organ dysfunction in the elderly.

Of 288 initially eligible patients (January to December 2020), 36 were excluded, forming a training cohort of 252 patients (67 MODSE, 185 non-MODSE) (Figure 1). An independent validation cohort of 60 patients was enrolled from January to March 2021.

### 2.3 Data collection and follow-up

The present study defines MODSE as concurrent or successive dysfunction of ≥2 organ systems following hip fracture surgery (Supplementary Table S1) (10).

#### 2.3.1 Perioperative data

Variables with potential predictive value for MODSE were collected through electronic health records (10, 12–21), including: preoperative variables (at admission), intraoperative parameters (surgery day: 7:00 AM to 7:00 AM next day), and postoperative biomarkers (samples were collected at 7 AM on the first day after surgery) were collected through electronic health records (Supplementary Table S2).

#### 2.3.2 Post-discharge follow-up

The 252 patients in the training cohort were prospectively followed for 24 months post-discharge via outpatient visits, telephonic consultations, and online communication. Observational endpoints included survival status, loss to follow-up, and all-cause mortality (non-hip fracture-related deaths, accidents, natural demise).

### 2.4 Statistical analysis

Patients were stratified by MODSE occurrence. Univariate logistic regression identified candidate predictors (*p* ≤ 0.05), followed by least absolute shrinkage and selection operator (LASSO) regularization to select variables while controlling for overfitting. A logistic regression model was constructed to estimate odds ratios (ORs) with 95% confidence intervals (CIs). Model calibration was assessed using the Hosmer-Lemeshow test, and discrimination via area under the receiver operating characteristic curve (AUROC). Mortality outcomes were evaluated using Kaplan–Meier curves and Cox proportional hazards models. Variables meeting the univariate threshold (*p* < 0.10) were incorporated into a multivariable Cox model. Clinical relevance (e.g., biological plausibility of MODSE-mortality association) and sample size considerations guided model refinement. Hazard ratios (HRs) with 95% CIs were estimated to quantify associations. The statistical analyses process and graphical representations were performed using R software version 4.2.3 (R Foundation for Statistical Computing, Vienna, Austria). *p*-values < 0.05 were considered to be statistically significant.

## 3 Results

In our study, all 252 patients underwent surgical treatment for hip fractures, including 107 femoral neck fractures treated with hip arthroplasty via the posterolateral approach (13 hemi-arthroplasties and 94 total hip arthroplasties) and 145 intertrochanteric fractures treated with proximal femoral nail fixation (114 standard intramedullary nails and 31 extended nails). There was no difference in the constituent ratio of surgical methods between the two groups (*p* = 0.054).

### 3.1 Clinical features for MODSE

Among the 252 subjects, 67 patients developed MODSE following hip fracture surgery, with 57 cases classified in the pre-failure stage and 10 cases progressing to the failure stage. The mortality rate within 30 days post-surgery was 0% for patients in the pre-failure stage of MODSE, while it reached 50% (5 out of 10) for those who progressed to the failure stage. The median number of affected organs or systems in patients with MODSE was 3 [interquartile range (IQR), 2 to 4]. The organs or systems primarily involved were the heart (59.7%), brain (59.7%), lung (58.2%), kidney (38.8%), gastrointestinal tract (29.9%), peripheral circulation (25.4%), coagulation function (20.9%), and liver (7.5%).

### 3.2 Univariate analysis of candidate variables

**Figure 2:**
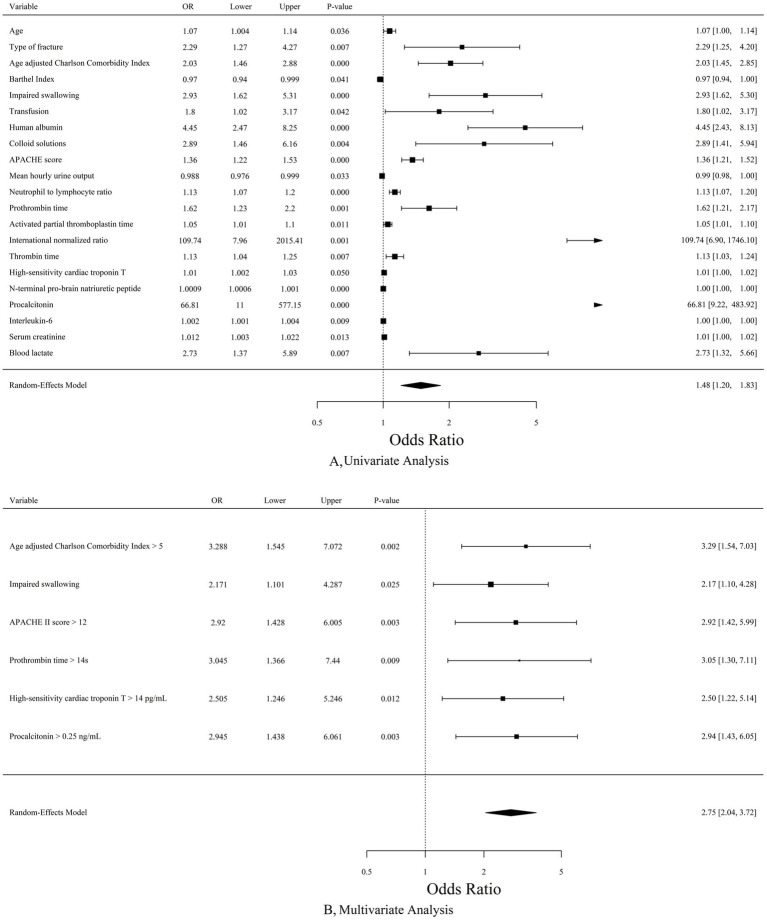
**(A)** Forest plot for variables associated with MODSE by univariate analysis. **(B)** Forest plot for the results of multivariate analysis by Lasso regression. MODSE, multiple organ dysfunction in the elderly; OR, odds ratio; APACHE, Acute Physiology and Chronic Health Evaluation.

Statistical descriptions of all candidate variables were provided in Supplementary Table S2. Figure 2A illustrated the results of univariate analysis for variables showing significant associations with MODSE.

### 3.3 Formulation and validation of clinical prediction model

The multivariate regression analysis revealed several factors strongly associated with postoperative MODSE. These included aCCI > 5 (OR = 3.288, 95%CI 1.545–7.072, *p* = 0.002), Impaired swallowing (OR = 2.171, 95%CI 1.101–4.287, *p* = 0.025), APACHE II score (“>12” vs. “9–12” and “≤8”, OR = 2.920, 95%CI 1.428–6.005, *p* = 0.003), PT > 14 s (OR = 3.045, 95%CI 1.366–7.440, *p* = 0.009), hs-cTnT > 14 pg./mL (OR = 2.505, 95%CI 1.246–5.246, *p* = 0.012) and PCT (“>0.25 ng/mL” vs. “0.11–0.25 ng/mL” and “≤0.10 ng/mL”, OR = 2.945, 95%CI 1.438–6.061, *p* = 0.003) (Figure 2B).

**Figure 3:**
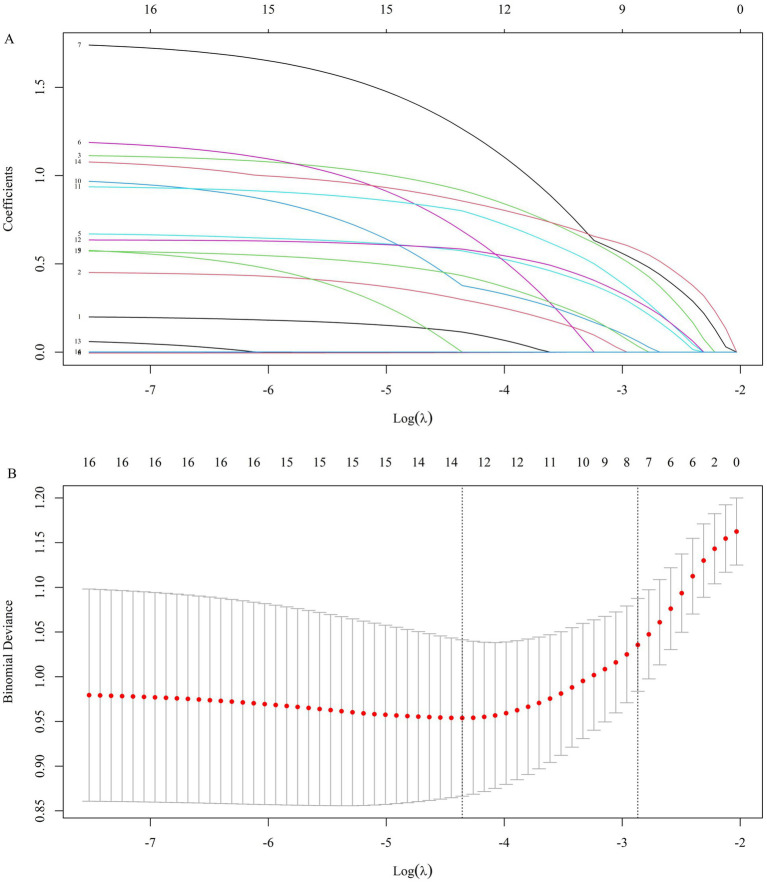
Coefficient path graph **(A)** and regression cross validation curve **(B)** of Lasso regression.

Figure 3A presents the coefficient trajectory plot of Lasso regression, where each line illustrates the variation trajectory of coefficients for independent variables. The X-axis denotes the logarithm of the regularization parameter (log(*λ*)), and the Y-axis represents the coefficient magnitude. Additionally, the cross-validation curve of Lasso regression was shown in Figure 3B, with the X-axis indicating the logarithm of the penalty parameter (log(λ)) and the Y-axis depicting the likelihood deviance metric.

Subsequently, the verification set’s independent variable data were inputted into the prediction model equation to determine the probability of MODSE occurrence, which was then compared with the observed outcomes. Calibration analysis showed a good agreement between predicted and observed MODSE probabilities, with a Hosmer-Lemeshow *p* value of 0.609 (Supplementary Figure S1). In terms of differentiation, the ROC curve demonstrated an AUROC of 0.81 (Supplementary Figure S2), indicating high accuracy of the prediction model in distinguishing MODSE from non-MODSE in elderly hip fracture patients. Supplementary Figure S3 illustrated that the LASSO model (red line) provides additional net benefit over the “All” and “None” strategies across most risk thresholds, highlighting its clinical utility over a wide range of thresholds.

### 3.4 Clinical outcomes

The median length of stay for all patients was 14.0 days (IQR, 11.0–19.0), which was significantly longer in the MODSE group (19.0 days, IQR 15.0–23.0) compared to the non-MODSE group (13.0 days, IQR 10.0–17.0) (*p* < 0.001). The 30-day mortality rate after surgery was 1.98%, with all deaths occurring exclusively in the MODSE group (7.5% in MODSE vs. 0% in non-MODSE group, *p* = 0.001) (Table 1).

**Table 1:** Clinical outcomes of MODSE and non-MODSE patients.

Outcome	Non-MODSE (*n* = 185)	MODSE (*n* = 67)	*p*-value	Crude HR (95% CI)	*P*-value	Adjusted HR (95% CI)	*P*-value
Length of stay (days)	13.0 (10.0, 17.0)	19.0 (15.0, 23.0)	<0.001				
Death within 30 days (Yes, %)	0 (0)	5 (7.5)	0.001				
Death within 1 year (Yes, %)	12 (6.5)^a^	16 (23.9)	<0.001	4.120 (1.948, 8.712)	<0.001	3.125 (1.417, 6.891)^b^	0.005
Death within 2 years (Yes, %)	23 (12.4)^c^	25 (37.3)	<0.001	3.560 (1.995, 6.352)	<0.001	2.876 (1.566, 5.280)^d^	0.001

a Five patients lost to follow-up.

b HR value was adjusted for age, age-adjusted Charlson Comorbidity Index (aCCI) and impaired swallowing.

c Nine patients lost to follow-up.

d HR value was adjusted for age, time-to-surgery, aCCI, and impaired swallowing.

MODSE, multiple organ dysfunction in the elderly; HR, hazard ratio; CI, confidence interval.

**Figure 4:**
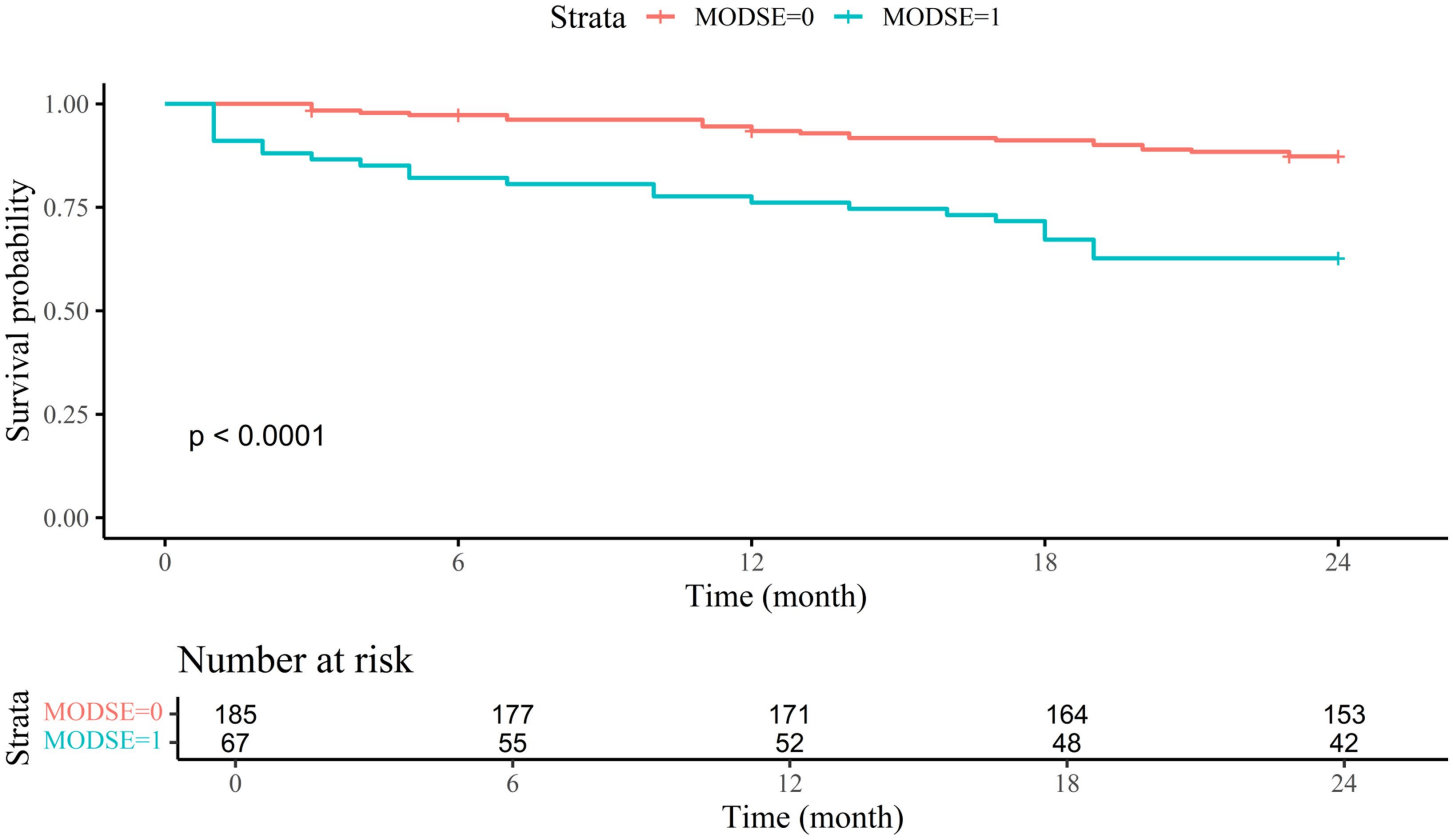
Survival curves for MODSE and non-MODSE patients MODSE, multiple organ dysfunction in the elderly.

Five patients were lost to follow-up in the first year after discharge, with an additional four patients lost in the second year, all belonging to the non-MODSE group. Within 1 year, 28 patients died, resulting in a 1-year mortality rate of 11.1%. The 1-year mortality rate was significantly higher in the MODSE group (23.9%) compared to the non-MODSE group (6.5%) [Adjusted hazard ratio (HR) = 3.125, 95% CI 1.417 to 6.891, *p* = 0.005] (Table 1; Figure 4). By the second year post-discharge, 48 patients had died, equating to a mortality rate of 19.0%. Among these, 23 patients (12.4%) were from the non-MODSE group, and 25 patients (37.3%) belonged to the MODSE group. The 2-year mortality rate for MODSE patients also significantly increased (Adjusted HR = 2.876, 95% CI 1.566 to 5.280, *p* = 0.001) (Table 1; Figure 4).

## 4 Discussion

Early surgical treatment is a standard practice in hip fracture management, yet there’s insufficient evidence on perioperative care (22). Postoperative risk factors logically have a greater impact on 30-day outcomes compared to preoperative factors (23). The majority of deaths within 30 days following hip fracture occurred in the hospital, predominantly within the first 10 days of admission (24). Our cohort study focused on prevalent systemic complications that occur following surgery. Specifically, we aimed to identify postoperative MODSE as a composite adverse outcome during hospitalization and determine its significance as the primary exposure factor for long-term mortality. MODSE entails dysfunction across eight organs or systems and is defined by clear diagnostic criteria (Supplementary Table S1) (9, 10). Besides rare or local complications, the diagnostic criteria for MODSE cover the majority of systemic complications typically observed following hip fracture surgery. Among our study population, MODSE incidence was 26.6% (67 out of 252), with 85.1% (57 out of 67) categorized as pre-failure cases, suggesting a relatively low threshold for diagnosing MODSE. Notably, mild or early organ function impairment is common in these patients, underscoring the need for early warning and multidisciplinary interventions. Distinct differences in clinical characteristics were observed between patients with and without MODSE throughout the hospitalization period. Patients with MODSE tended to be older, with a higher proportion experiencing intertrochanteric fracture and exhibiting significantly elevated aCCI. Moreover, they demonstrated lower Barthel Index and greater swallowing impairment. On the day of surgery, patients with MODSE received higher volumes of fluids, while their hourly urine output markedly decreased. Subsequently, laboratory results obtained on the first postoperative day revealed signs of multiple organ damage in these patients. The above findings show that systemic pathophysiological changes following hip fracture and/or surgery in the elderly may involve ongoing processes affecting multiple organs or systems. These changes can manifest as either acute dysfunction or exacerbations of pre-existing chronic organ impairments.

The multivariate analysis revealed that the aCCI, impaired swallowing, and early postoperative APACHE II scores synergistically delineate a pathological continuum from chronic vulnerability to acute multi-organ dysfunction in elderly hip fracture patients. Elevated aCCI scores, reflecting pre-existing multimorbidity and diminished physiological reserve, establish a baseline susceptibility to systemic decompensation. In our study, this susceptibility is further substantiated by its direct correlation with MODSE and alignment with prior research demonstrating associations between comorbidities, skeletal complications, and increased mortality risk (25–27). This chronic frailty is exacerbated by acute physiological insults captured through APACHE II scoring within 24 h post-surgery, which not only quantifies immediate postoperative instability but also aligns with emerging evidence of its prognostic utility in optimizing surgical timing and complication mitigation (28). Impaired swallowing, a prevalent issue among frail elderly individuals, may serve as a critical nexus linking these domains: it significantly heightens the risk of aspiration, malnutrition (29), and pulmonary interleukin-6 mediated inflammatory cascades (30, 31), potentially escalating localized respiratory compromise into systemic cytokine storm syndromes. From a clinical perspective, incorporating APACHE II scoring alongside dysphagia screening within the first 24 h postoperatively could provide a strategic approach for early identification of high-risk patients.

Our model identifies PCT, PT and hs-cTnT as independent predictors, reflecting the trauma-induced nexus of inflammatory, coagulopathic (32, 33), and cardiovascular cascades that drive multi-organ dysfunction. Elevated PCT levels (>0.25 μg/L) on the first postoperative day, even below conventional sepsis thresholds, signify subclinical inflammation capable of triggering organ dysfunction, consistent with evidence that minor PCT rises (>0.39 μg/L) predict mortality in orthopedic cohorts (34). Concurrently, PT abnormalities revealed a dual thrombotic-hemorrhagic tendency, likely driven by fracture-induced tissue factor release (35), preoperative polypharmacy, and vitamin K deficiency (36, 37). Crucially, extending our prior finding that admission hs-cTnT predicts MODSE risk (38), this study demonstrates that postoperative Day 1 hs-cTnT elevation retains prognostic significance. These findings highlight the importance of early postoperative biomarker monitoring, even within subcritical ranges, to guide timely preemptive interventions.

The progression from hip fracture to death often involves the development of vital organ failure, particularly evident in patients who experience early mortality post-fracture. In this study, patients who progressed to the MODSE failure stage exhibited a 30-day mortality rate of up to 50%. Postoperative MODSE was also significantly associated with long-term mortality. The 1-year mortality rate following hip fracture in mainland China is lower than in many other countries (12). Our study demonstrated a more favorable 1-year mortality rate of 11.1%. Even so, developing MODSE post-hip fracture surgery substantially increased both 1-year and 2-year mortality rates by 3.13-fold and 2.88-fold, respectively. Prior studies have shown that frail elderly individuals with hip fractures, managed non-surgically, face significantly heightened long-term mortality due to complications across multiple organs or systems (39, 40). Consequently, whether treated surgically or not, the high 30-day and long-term mortality rates associated with hip fracture can be primarily linked to organ dysfunction. Perioperative management of hip fracture in the elderly prioritizes the preservation of vital organ function, with early restoration of limb function aimed at averting organ failure and mortality.

The study has several limitations. Firstly, in this study, we strictly followed the published diagnostic standards of MODSE (Supplementary Table S1) to divide patients into two groups. All research team members received unified training before study beginning, and used same case report forms to ensure the criteria applied consistently. But we deeply realize that external validation is still lacking, this is because our current research mainly focused on clinical application. Moreover, the factors contributing to poor outcomes after hip fracture surgery are multifaceted and complex, certain variables that potentially influence postoperative MODSE may still have been omitted. Second, this research is a single-center observational study with a limited sample size, which inevitably introduces potential biases. Finally, due to some patients passing away at home rather than in a medical facility, their families were unable to provide an accurate cause of death. Consequently, further analysis regarding the cause of death could not be conducted in this study.

## 5 Conclusion

The occurrence of postoperative MODSE in elderly patients with hip fracture is not uncommon. Accurate prediction of MODSE is crucial and feasible because it not only represents a poor outcome during hospitalization but also correlates with prolonged hospital stay, increased 30-day mortality rates following surgery, as well as higher mortality rates at 1 and 2 years after discharge.

## Data Availability

The datasets for this study is available from the corresponding author (Email: qin18716111836@126.com) on reasonable request.
