# Supplementary material for: Clinical prediction model and 2-year mortality for multiple organ dysfunction in patients aged 80 years or older following hip fracture surgery: a prospective cohort study
Source: Front Med (Lausanne). 2025 Jul 25;12:1515557. doi: 10.3389/fmed.2025.1515557 (PMC12331632; doi:10.3389/fmed.2025.1515557)
Supplement: Supplementary file 1 [file Table_1.docx]

**Supplementary Table 1 Diagnostic criteria for MODSE†**

|  | **Pre-failure stage** | **Failure stage** |
| --- | --- | --- |
| Heart ‡ | i. Emerging arrhythmia, normal cardiac enzymes ii. Exertional dyspnea, no definite signs of heart failure iii. Increased PAWP (13 ~ 19mmHg) § | i. Reduced stroke volume (EF ≤ 40%) ii. PAWP ≥ 20mmHg ¶ iii. Definite signs and symptoms of heart failure |
| Lung | i. PaCO_2_ 45 ~ 49mmHg ii. SaO_2_ < 90% iii. pH 7.30 ~ 7.35 or 7.45 ~ 7.50 iv. 200mmHg < PaO_2_/FiO_2_ ≤ 300mmHg v. No MV requirement | i. PaCO_2_ > 50mmHg ii. SaO_2_ < 80% iii. pH < 7.30 iv. PaO_2_/FiO_2_ ≤ 200mmHg v. MV requirement |
| Kidney | i. Decreased UOP (20 ~ 40mL/h), good response to diuretics ii. Scr 177.0 ~ 265.2μmol/L (or > 20% increase from baseline) iii. No dialysis requirement | i. Decreased UOP (< 20mL/h) and poor response to diuretics ii. Scr > 265.2μmol/L (or > 20% increase from baseline) iii. Dialysis requirement |
| PC | i. Decreased UOP (20-40mL/h) ii. MAP 50 ~ 60mmHg or > 20% decrease from baseline, good response to vasopressors iii. Exclude hypovolemia | i. Decreased UOP (< 20mL/h) complicated with cold limbs and cyanosis ii. MAP < 50mmHg, multiple vasopressors and inotropic agents dependence iii. Exclude hypovolemia |
| Liver | 1. TBIL 35 ~ 102μmol/L ii. ALT elevated < 2 × normal value iii. Markedly increased bilirubin with normal or decreased transaminases | i. TBIL ≥ 102μmol/L ii. ALT elevated > 2 × normal value iii. Hepatic encephalopathy |
| GT | 1. Abdominal distension ii. Hypoactive bowel sounds iii. Acalculous cholecystitis | 1. Severe abdominal distension, disappeared bowel sounds ii. Stress ulceration complicated bleeding or perforation iii. Necrotizing enteritis v. Spontaneous gallbladder perforation |
| CNS | i. Obtundation ii. Disorientation iii. GCS 9 ~ 12 | i. Diffuse neurologic injury ii. No response to speech or voice iii. No response to pain v. GCS ≤ 8 |
| CS | i. PLT 51 ~ 99 × 10^9^/L ii. FIB ≥ 2 ~ 4g/L iii. PT and TT prolonged < 3s iv. D-dimer increased < 2 × normal value v. No obvious signs of bleeding | i. PLT ≤ 50 × 10^9^/L with decreasing trend ii. FIB < 2g/L iii. PT and TT prolonged > 3s iv. D-dimer increased > 2 × normal value v. Obvious bleeding |

Note: † If two or more organ functions meet the criteria for pre-failure stage while other organ functions are normal, a diagnosis of MODSE pre-failure stage is made. If two or more organ functions meet the criteria for failure stage while other organ functions are normal or in the pre-failure stage, a diagnosis of MODSE failure stage is made. Two or more abnormal values are required for the diagnosis of each criterion. ‡ The criterion of PAWP is replaced by LUS findings, § Replaced by ≤ 30 B-lines on 28 zone LUS, ¶ Replaced by > 30 B-lines on 28 zone LUS.

Abbreviations: PAWP, pulmonary artery wedge pressure; EF, ejection fraction; PaCO_2_, partial pressure of carbon dioxide; SaO_2_, arterial oxygen saturation; PaO_2_, arterial partial pressure of oxygen; FiO_2_, inspired oxygen concentration; MV, mechanical ventilation; UOP, urine output; Scr, serum creatinine; PC, Peripheral circulation; MAP, mean arterial pressure; TBIL, total bilirubin; ALT, alanine aminotransferase; GT, gastrointestinal tract; CNS, central nervous system; GCS, Glasgow score; CS, coagulation system; PLT, platelet; FIB fibrinogen; PT, prothrombin time; TT, thrombin time; LUS, lung ultrasound.
