# Supplementary material for: Clinical prediction model and 2-year mortality for multiple organ dysfunction in patients aged 80 years or older following hip fracture surgery: a prospective cohort study
Source: Front Med (Lausanne). 2025 Jul 25;12:1515557. doi: 10.3389/fmed.2025.1515557 (PMC12331632; doi:10.3389/fmed.2025.1515557)
Supplement: Supplementary file 2 [file Table_2.docx]

**Supplementary Table 2**

Comparison of all candidate variables between MODSE and non-MODSE groups

| Variables | | Non-MODSE (n=185) | MODSE (n=67) | *P*-value ^a^ |
| --- | --- | --- | --- | --- |
| **Preoperative basic characteristics** | | | | |
| Age (years) | | 85.0 (82.0, 89.0) | 86.0 (84.0, 91.0) | 0.036 |
| Gender (Male/Female) | | 49 (26.5)/136 (73.5) | 22 (32.8)/45 (67.2) | 0.323 |
| Type of fracture (Femoral neck/intertrochanteric) | | 88 (47.6)/97(52.4) | 19 (28.4)/48 (71.6) | 0.007 |
| Operative approach | total hip arthroplasty | 77 (30.6) | 17 (6.7) | 0.054 |
|  | hemi-arthroplasty | 11 (4.4) | 2 (0.8) |  |
|  | PFNA (standard intramedullary nail) | 77 (30.6) | 37 (14.7) |  |
|  | PFNA (extended intramedullary nail) | 20 (7.9) | 11 (4.4) |  |
| Site of fracture (Left/Right) | | 100 (54.1)/ 85 (45.9) | 18 (49.3)/65 (50.7) | 0.500 |
| Age-adjusted Charlson Comorbidity Index | | 5.0 (4.0, 5.0) | 5.0 (4.0, 6.0) | < 0.001 |
| Barthel Index | | 35.0 (30.0, 40.0) | 35.0 (30.0, 40.0) | 0.041 |
| Impaired swallowing (Yes,%) | | 42 (22.7) | 31 (46.3) | < 0.001 |
| Time-to-surgery (days) | | 6.0 (4.0, 8.0) | 7.0 (5.0, 9.0) | 0.095 |
| **Variables on the day of surgery ^b^** | | | | |
| Method of anesthesia (General /Regional) | | 181 (97.8)/4 (2.2) | 66 (98.5)/1 (1.5) | 0.738 |
| Operative duration (min) | | 50.0 (40.0, 60.0) | 53.0 (40.0, 67.0) | 0.160 |
| Estimated intraoperative blood loss (mL) | | 120.0 (100.0,180.0) | 120.0 (100.0,180.0) | 0.836 |
| Postoperative analgesia (PCIA/PCNA) | | 44 (23.8)/141 (76.2) | 18 (26.9)/49 (73.1) | 0.616 |
| Transfusion (Yes, %) | | 70 (37.8) | 35 (52.2) | 0.042 |
| Human albumin (Yes, %) | | 61 (33.0) | 46 (68.7) | < 0.001 |
| Colloid solutions (Yes, %) | | 118 (63.8) | 56 (83.6) | 0.004 |
| APACHEⅡ score | | 10 (8,12) | 12 (10,14) | < 0.001 |
| Positive fluid balance (mL) | | 2050.0 (1520.0, 2565.0) | 2270.0 (1750.0, 2920.0) | 0.572 |
| Mean hourly urine output (mL) | | 59.0 (42.4, 81.4) | 51.4 (35.0, 67.5) | 0.033 |
| **Laboratory findings ^c^** | | | | |
| White blood cell count (×10^9^ per L) | | 8.2 (6.6, 9.9) | 7.7 (6.2, 9.7) | 0.966 |
| Neutrophil-to-lymphocyte ratio | | 7.2(4.9, 9.8) | 9.0 (6.6, 14.0) | < 0.001 |
| Red blood cell count (×10^9^ per L) | | 3.3 (3.0, 3.6) | 3.4 (3.0, 3.7) | 0.885 |
| Haemoglobin (g/L) | | 100.4 ± 12.3 | 101.7 ± 14.0 | 0.458 |
| Platelet count (×10^9^ per L) | | 157.0 (117.5, 201.0) | 172.0 (116.0, 226.0) | 0.124 |
| Prothrombin time (s) | | 14.3 (13.7, 14.8) | 14.7 (14.3, 15.4) | 0.001 |
| Activated partial thromboplastin time (s) | | 40.1 (37.1, 45.4) | 41.9 (38.7, 48.1) | 0.011 |
| International normalized ratio | | 1.18 (1.13, 1.24) | 1.23 (1.18, 1.30) | 0.001 |
| Thrombin time (s) | | 16.4 (15.5, 18.1) | 17.4 (15.9, 19.0) | 0.007 |
| Fibrinogen (g/L) | | 4.0 ± 0.9 | 3.9 ± 0.8 | 0.391 |
| D-dimer (mg/L) | | 2.6 (1.7, 5.0) | 3.4 (2.2, 5.7) | 0.299 |
| Fibrin (ogen) degradation products (mg/L) | | 8.4 (5.1, 17.1) | 11.0 (6.8, 22.1) | 0.665 |
| High-sensitivity cardiac troponin T (pg/mL) | | 14.0 (10.0, 22.5) | 22.0 (15.0, 35.0) | 0.050 |
| N-terminal pro-brain natriuretic peptide (ng/L) | | 583.9 (327.3,1052.0) | 1306.0 (787.8, 2471.0) | < 0.001 |
| Procalcitonin (ng/mL) | | 0.13 (0.09, 0.20) | 0.23 (0.14, 0.44) | < 0.001 |
| Interleukin-6 (pg/mL) (1 missing) | | 139.2 (86.3, 204.0) | 148.3 (92.2, 260.5) | 0.009 |
| Alanine aminotransferase (U/L) | | 16.0 (11.0, 25.0) | 16.0 (10.0, 23.0) | 0.428 |
| Total bilirubin (μmol/L) | | 12.1 (9.0, 17.2) | 11.3 (8.0, 16.9) | 0.857 |
| Prealbumin (mg/L) | | 117.0 ± 33.6 | 110.6 ± 30.7 | 0.172 |
| Albumin (g/L) | | 29.7 ± 3.0 | 30.5 ± 3.8 | 0.098 |
| Serum creatinine (μmol/L) | | 60.0 (47.0, 78.0) | 68.0 (51.0, 94.0) | 0.013 |
| Cystatin C (mg/L) | | 1.2 (1.0, 1.5) | 1.3(1.1, 1.9) | 0.425 |
| Uric acid (μmol/L) | | 237.0 ± 94.3 | 241.6 ± 95.7 | 0.788 |
| Fasting blood glucose (mmol/L) | | 5.9 (5.2, 6.9) | 5.8 (4.9, 7.0) | 0.490 |
| Serum potassium (mmol/L) | | 4.1 ± 0.4 | 4.2 ± 0.5 | 0.250 |
| Serum sodium (mmol/L) | | 139.1 ± 3.3 | 139.1 ± 4.2 | 0.950 |
| Serum chlorine (mmol/L) | | 103.0 ± 3.6 | 102.7 ± 4.5 | 0.582 |
| Serum calcium (mmol/L) | | 2.0 ± 0.1 | 2.0 ± 0.1 | 0.475 |
| Serum phosphorus (mmol/L) | | 1.0 ± 0.2 | 1.1 ± 0.2 | 0.412 |
| Oxygenation index (mmHg) | | 281.0 (242.0, 357.0) | 275.0 (215.2, 315.2) | 0.071 |
| Partial pressure of arterial carbon dioxide (mmHg) | | 42.0 (39.0, 47.0) | 43.0 (39.0, 46.0) | 0.360 |
| Standard bicarbonate (mmol/L) | | 25.1 (24.0, 26.7) | 24.4 (22.9, 26.5) | 0.397 |
| Blood lactate (mmol/L) | | 1.0 (0.9, 1.2) | 1.1 (0.9, 1.5) | 0.007 |

Notes: Data are presented as number (%) or mean ± standard deviation or median (interquartile range).

^a^ Based on the analysis of univariate logistic regression; ^b^ Defined as the interval from 7:00 am on the day of surgery to 7:00 am on the following day; ^c^ Laboratory results were obtained from specimens collected around 7:00 am on postoperative day 1.

Abbreviations: MODSE, multiple organ dysfunction in the elderly; PFNA, proximal femoral nail fixation; PCIA, patient-controlled intravenous analgesia; PCNA, patient-controlled nerve analgesia; APACHE, Acute Physiology and Chronic Health Evaluation.
